# Anesthesia methods for full-endoscopic lumbar discectomy: a review

**DOI:** 10.3389/fmed.2023.1193311

**Published:** 2023-08-17

**Authors:** Bin Zheng, Chen Guo, Shuai Xu, Haoyuan Li, Yonghao Wu, Haiying Liu

**Affiliations:** Spine Surgery, Peking University People’s Hospital, Beijing, China

**Keywords:** full-endoscopic lumbar discectomy, general anaesthesia, local anesthesia, epidural anesthesia, erector spine plane block

## Abstract

Full-endoscopic lumbar discectomy under local anesthesia is major trends for the treatment of lumbar disc herniation in spine minimally invasive surgery. However, sometimes local anesthesia is not enough for analgesic in surgery especially in interlaminar approach. This study summarizes the current study of anesthesia methods in full-endoscopic lumbar discectomy. Local anesthesia is still the most common anesthesia method in full-endoscopic lumbar discectomy and the comparison group for other anesthesia methods due to high safety. Compared to local anesthesia, Epidural anesthesia is less applied in full-endoscopic lumbar discectomy but reports better intraoperative pain control and equivalent safety due to the motor preservation and pain block characteristic of ropivacaine. General anesthesia can achieve totally pain block during surgery but nerve injury can not be ignored, and intraoperative neuromonitoring can assist. Regional anesthesia application is rare but also reports better anesthesia effects during surgery and equivalent safety. Anesthesia methods for full-endoscopic lumbar discectomy should be based on patient factors, surgical factors, and anesthesiologist factors to achieve satisfactory anesthesia experience and successful surgery.

## Introduction

Lumbar disc herniation is a prevalent diseases among all population, as evidenced by previous studies (1). The selection of appropriate treatment for individual patient remains hotspots. Traditional open spine surgery reports definite efficacy, but has disadvantages of lengthy operation time, large bleeding volume, high rates of complications and postoperative back pain (2, 3). Since Yeung first reported the Yeung endoscopic spine system under local anesthesia (4), full-endoscopic lumbar discectomy has become a mature and mainstream minimally invasive surgical technique for treating lumbar disc herniation.

Most surgeons apply local anesthesia in full-endoscopic lumbar discectomy surgery. Under local anesthesia, patients are awake during the surgery so that surgeon can identify intraoperative neurologic injury through sensory and motor feedback from patients. However, local anesthesia may not always provide adequate analgesia. Previous studies have reported severe pain occurs particularly during needle puncture, foraminoplasty, and discectomy (5). Uncontrolled severe pain leads to hemodynamical fluctuation，unsatisfactory surgery experience and higher rates of complications. Therefore, finding a suitable anesthesia method for full-endoscopic lumbar discectomy would have great clinical significance for successful surgery. The aim of the current review is to summarize studies of anesthesia method for full-endoscopic lumbar discectomy.

### Local anesthesia

Local anesthesia is the most commonly applied anesthetic method in full-endoscopic lumbar discectomy since Yeung first reported in full-endoscopic lumbar discectomy surgery study (4). Surgeon injects local anesthetic layer-by-layer to achieve infiltration from the skin, muscle to the surgical site (6). This effectively numbs targeted area and reduces pain during surgery. In cases when the surgical anatomy under endoscopy is challenging to distinguish, surgeons can identify nerve through feedback from awake patients to avoid injury (7).

However, despite its safety, the anesthesia efficacy is not always adequate enough to achieve satisfactory analgesic effects, particularly during conducting working channel, arthroplasty of articular process or foramen, and discectomy. Besides transforaminal endoscopic lumbar discectomy (TELD), interlaminar endoscopic lumbar discectomy (IELD) is another approach in full-endoscopic lumbar discectomy, which is superior in L5-S1 disc herniation and high grade axillary herniated disc (8). More muscle retraction, inevitable spinal cord nerve and dura sacs pulling lead to violent pain during IELD and more surgeons choose to apply general anesthesia in IELD surgery to achieve satisfactory anesthesia efficacy (9). But there is a risk of nerve root injury caused by using nucleus pulposus forceups, electrical coagulation and other surgical procedures. Patients experiencing intolerable pain may require a change in anesthesia method or even the termination of surgery (10, 11).

In order to fill pain defect gap, Intravenous sedation combined with local anesthesia is a common approach to ensure patient comfort and safety. The level of sedation can be adjusted to ensure that the patient is responsive but comfortable throughout the surgery. Commonly used sedative agents include midazolam, propofol, and fentanyl. Dexmedetomidine (DEX), a new-generation, highly selective α2 adrenergic receptor agonist, is widely used in spine surgery due to its efficacy to achieve a balance between an effective analgesia and sedation regimen, while having minimal effect on the respiratory system. In some study, adding DEX is a feasible method to comprise the analgesic defects under local anesthesia. It can also provide a more stable level of sedation, allowing the patient to be awake and cooperative during the procedure. Hypotension/bradycardia and delayed emergence are major shortcomings of dexmedetomidine, which can be prevented by ketamine intraoperatively. Studies have shown that adding DEX is a feasible method to address the analgesic defects associated with local anesthesia (12–15).

Opioids are a class of analgesic drugs that exert their effects by binding to specific receptors in the brain, spinal cord, and other parts of the body. In full-endoscopic lumbar discectomy, opioids are sometimes used to alleviate pain, particularly when other analgesic methods such as local anesthesia and DEX are not effective (16). However, the use of opioids in full-endoscopic lumbar discectomy surgery is not without risks. Opioids can cause respiratory depression, nausea, vomiting, constipation, and other side effects (17, 18). Proper multiple-anesthesia strategy can achieve better intraoperative pain control and less opioids usage to improve anesthesia effects during surgery.

### Epidural anesthesia

Epidural is a commonly used anesthesia method in surgery, particularly in delivery and lower limbs surgeries. During epidural anesthesia, anesthetic is injected into the epidural space to block the spinal nerve root to achieve anesthesia effects. Furthermore, anesthesiologist can implant epidural catheter to adjust anesthesia plane, anesthesia time and dose (19, 20).

Epidural anesthesia can achieve better anesthesia effects during surgery, as common nerve conduction pathway toward spine are blocked. However relevant studies on application of epidural in full-endoscopic lumbar discectomy is rare, which might be attributed to complex anesthesia procedure and unique complications.

The motor-sensory separation characteristic of ropivacaine under certain concentration can be effective in effective in surgery procedure, especially during require precise movements or muscle control, such as orthopedic surgery or hand surgery. Under ideal epidural anesthesia, pain is blocked but motor function is preserved, thus helping prevent nerve injury (21). Different nerve fibers have varyring susceptibilities to local anesthetics, mainly depends on diameter of nerves and myelination. Local anesthetics work by blocking the transmission of nerve impulses, and they do this by binding to specific sites on the sodium channels in nerve fibers. A fibers are the largest and most heavily myelinated fibers. The subgroup A fibers can be divided A-α, A-β, A-γ B and A-δ. Motor function is achieved by A-α、A-β、A-γ nerves (6–20 mm). A-δ fibers are smaller (1–5 mm) and less heavily myelinated, which are responsible for fast pain signals localized to a specific area. B fibers are smaller (1–3 mm) and less heavily myelinated than A nerves, which are primarily responsible for transmitting signals of the autonomic nervous system, such as the functions of the cardiovascular system, digestive system, and respiratory system. C fibers are the smallest (<1 mm) and least myelinated fibers, and they are responsible for carrying pain and temperature sensation. Compared to quick and sharp pain signals, C nerves transmit dull, poorly localized pain signals. Motor function is achieved by A-α, A-β, A-γ nerves (6–20 micrometers), and pain is mainly transmitted by myelinated A δ fibers and unmyelinated C fibers. Due to diameter difference and the characteristic of myelin sheath, C and A δ nerve fiber are more susceptible to local anesthetics than A-α, A-β, A-γ fiber, which leads to pain block and motor function preservation (22). The extent of motor function preservation and pain block can vary depending on the concentration and application of ropivacaine, as well as individual patient factors. The reasonable concentration of ropivacaine is 0.4% for pain block and motor preservation, and it provides satisfactory anesthesia effects and stable circulation (23).

Clinical studies and relevant meta-analysis report epidural anesthesia can achieve lower visual analogue scale (VAS) during surgery and better anesthesia satisfactory. There are no significant intergroup differences in the postoperative Oswestry Disability Index, and complication rates, indicating equivalent future effects between epidural anesthesia method and local anesthesia method (24–27). Therefore, epidural anesthesia can be regarded as an effective and safe anesthesia method for full-endoscopic lumbar discectomy. However, there still needs more high quality evidence to support.

### General anesthesia

Under general anesthesia, pain is completed blocked and patients are unconscious during surgery. This is achieved by administering a combination of intravenous drugs and inhaled anesthetic gases. General anesthesia in full-endoscopic lumbar discectomy might be applied in certain situations, including intolerable pain under local anesthesia, severe hemodynamic fluctuation or other situations. However, general anesthesia may carries additional risks such as airway and breathing complications, vomiting and longer recovery time etc. There are also increase risks of nerve injury, especially for inexperience spine surgeons,due to lack of instant feedback from patients (28).

As pain is totally blocked and patients are unconscious during surgery, there are no pain relevant comparison during surgery between general anesthesia and other anesthesia methods. Therefore, comparative study focus on complications, postoperative pain and future efficacy (29, 30). Compared to local anesthesia, overall complication rates are higher in general anesthesia groups due to better pain control. The risk of injury is closely related to the operator’s experience, proficiency and surgical sites. Mooney reported meta-analysis including 68 studies of total 5,269 patients (28). This study can be regarded as comprehensive review on comparison between local anesthesia and general anesthesia in full-endoscopic lumbar discectomy. For instant improvement, LA leads to overall improvements but GA indicates only VAS leg score improvement compared to preoperative score. Under general anesthesia, more extensive nerve traction may lead to increased pain after surgery. Full-endoscopic lumbar discectomy under general anesthesia can achieve better life quality after surgery due to the complete pain block, which allows for adequate operate nerve roots for complete discectomy or decompression (28). The number of studies comparing general anesthesia and other anesthesia is limited. Ren reports no difference between general anesthesia and epidural anesthesia in efficacy and safety. However, Ren still recommends epidural anesthesia for inexperience surgeons for better safety (31).

As surgical site is proximal to the nerves, and any iatrogenic damage could result in further pain, weakness, or other complications. Neuromonitoring can be a valuable assistance for monitoring the function of the nerves and spinal cord and to detect any potential damage or complications during the procedure, especially general anesthesia. Several neuromonitoring techniques can be used during full-endoscopic lumbar discectomy, including electromyography (EMG), somatosensory evoked potentials (SSEPs), and motor evoked potentials (MEPs). EMG measures the electrical activity of the muscles surrounding the spine, while SSEPs and MEPs measure the electrical signals generated by the spinal cord and nerves in response to sensory or motor stimulation (32–34). However, there lacks studies on INOM in full-endoscopic lumbar discectomy. This area can be further studied for higher safter of general anesthesia in full-endoscopic lumbar discectomy.

### Regional anesthesia

Spine plane block has been applied in postoperative analgesic in traditional spine surgery and reported satisfactory efficacy. Erector spinae plane block (ESPB) is a common regional anesthesia technique that injecting local anesthetic into the fascial plane surrounding the erector spinae muscles. The dorsal spine nerves are blocked to achieve anesthesia effects: by targeting the nerves responsible for transmitting pain from the surgical area, the ESP block can help reduce postoperative pain and potentially decrease the need for opioids (35). Due to satisfactory postoperative analgesic efficacy, EPSB can be applied as anesthesia method and reduce the need for opioid medications compared to local anesthesia. ESPB primarily blocks sensory nerves, and it is less likely to cause motor blockade A study published in the Chinese Journal of Anesthesiology reported better anesthesia effects, satisfactory effects and no major complications related to ESP block in 30 patients who underwent full-endoscopic lumbar discectomy. As for efficacy during surgery, Wu reports ESPB can achieve better pain control, less opioid consumption and better anesthesia satisfactory in patients undergoing full-endoscopic lumbar discectomy. And there is no difference in future life quality between erector spine plane block and local anesthesia (36). However, more high-quality studies are needed to confirm these findings and to determine the optimal dosage and timing of ESPB in full-endoscopic lumbar discectomy. Benefits and risks compared to other types of anesthesia, such as general or local anesthesia with sedation, are still being studied. Besides, ESPB is a relatively new technique and requires skill and experience to perform accurately, especially when using ultrasound guidance for Anesthesiologist.

Besides, there are other plane block such as thoracic-lumbar plane block or retrolaminar block. However, relevant studies lack for other plane block technique in full-endoscopic lumbar discectomy.

Ultrasound can be applied as guided tool for full-endoscopic lumbar discectomy. Zhang reports equivalent puncture, cannulation and operation times, and less X-ray exposure (37). Wu reports decreased radiation exposure and no serious complications (38). Since plane block is performed under ultrasound, there is feasibility in combining plane block and puncture guide. Surgeons can achieve analgesia and locate targeted areas to achieve better clinical results.

### Current evidence

The majority of the study we have assessed thus far in this field primarily relies on retrospective studies. Nevertheless, it is critical to acknowledge that these studies provide a somewhat limited level of evidence. In an effort to derive a more encompassing understanding, we have scrutinized the latest randomized controlled trials which focus on comparing anesthesia methods utilized in full-endoscopic lumbar discectomy. The initial parameters and the corresponding clinical outcomes gleaned from our rigorous review are succinctly encapsulated in Tables 1, 2 (27, 30, 39–43).

**Table 1:** Characteristic table of randomized controlled trials comparing anesthesia methods in full-endoscopic lumbar discectomy.

	Anesthesia methods	Samples	Operative time (minutes)	VAS leg pain	VAS back pain
Chen et al. (39)	GA/LA	50/73	74.78 ± 17.65/67.07 ± 33.87	74.35 ± 18.82/77.14 ± 18.02	60.43 ± 16.66/55.36 ± 20.27
Wang et al. (40)	EA/LA	46/46			
Zhu et al. (27)	EA/LA	46/47	80.89 ± 8.65/63.19 ± 9.93	7.31 ± 1.41/7.27 ± 1.69	2.83 ± 1.59/2.64 ± 1.55
Xu et al. (41)	EA/LA	49/46	64.91 ± 24.981/118.71 ± 24.598	8.23 ± 2.054/8.71 ± 1.954	5.20 ± 3.15/4.88 ± 2.725
Wu et al. (42)	GA/LA	50/48	70.7 ± 35.3/78.5 ± 33.1	7.4 ± 0.6/7.4 ± 0.5^1^	
Ye et al. (30)	GA/LA	30/30	78.64 ± 19.12/50.12 ± 23.65	*p* > 0.05	P > 0.05
Zhang et al. (43)	EA/LA	100/100	143.56 ± 15.53/ 147.18 ± 19.67	6.27 ± 1.08/ 6.41 ± 1.31	4.17 ± 1.31/3.98 ± 1.24

GA, general anesthesia; LA, local anesthesia visual; VAS, analogue scale.

^1^The study does not specifically report whether it is VAS leg pain or VAS back pain.

**Table 2:** Clinical outcomes of randomized controlled trials comparing anesthesia methods in full-endoscopic lumbar discectomy.

	Method	VAS leg pain intraoperative	VAS back pain intraoperative	VAS leg pain last follow up	VAS back pain last follow up	Complications
Chen et al. (39)	GA/LA			11	10.4	13/10
Wang et al. (40)	EA/LA			3.13 ± 1.12/3.26 ± 1.17^1^		6/20
Zhu et al. (27)	EA/LA	2.47 ± 0.51/6.87 ± 0.51	3.62 ± 0.55/7.86 ± 0.59	2.02 ± 0.76/3.06 ± 0.73	1.01 ± 0.22/1.47 ± 0.27	9/2
Xu et al. (41)	EA/LA	1.38 ± 1.484/5.67 ± 1.883	1.25 ± 1.164/4.67 ± 1.183	1.25 ± 1.643/1.57 ± 1.579	1.18 ± 0.811/1.93 ± 0.712	
Wu et al. (42)	GA/LA			1.6 ± 0.7*/1.4 ± 0.6^1^		4/9
Ye et al. (30)	GA/LA			*P* > 0.05^2^	*P* > 0.05^2^	7/2
Zhang et al. (43)	EA/LA	*p* < 0.05^1,2,3^		/	/	0

GA, general anesthesia; LA, local anesthesia; VAS, analogue scale.

^1^The study does not specifically report whether it is VAS leg pain or VAS back pain.

^2^The study provides bar chart without detailed data.

^3^Epidural anesthesia is superior to local anesthesia in VAS score.

### Ideal clinical trial model

Previous clinical trials presented different clinical design and the quality of each study diverse. The difference between studies focus on the outcomes, and we have summarized valuable outcomes for future study.

According to Cochrane criteria, we have designed a reasonable PICO model for future study on investigating anesthesia methods in full-endoscopic lumbar discectomy.

P: Patients diagnosed with lumbar disc herniation are included. Patients with lumbar canal stenosis, spondylolisthesis, infection, tumor, malformation and tuberculosis are excluded.

I: Patients underwent full-endoscopic lumbar discectomy (including TELD or IELD) surgery under other anesthesia methods. Anesthesia methods should be standardized and administered by experienced anesthesiologists to minimize variations in outcomes.

C: Patients underwent full-endoscopic lumbar discectomy (including TELD or IELD) surgery under local anesthesia. Anesthesia methods should be standardized and administered by experienced surgeons to minimize variations in outcomes.

O: Primary Outcomes: Intraoperative Vas: (I) Max Vas during puncture and conducting work channel. (II) Max Vas during foraminoplasty. (III) Max Vas during discectomy. (IV) Anesthesia satisfactory rates. (V) Complications during surgery. (VI) The number of surgery exit and change anesthesia methods, and the details should be recorded.

Secondary outcomes: (I) postoperative complications. (II) 1-day Vas back pain after surgery. (III) 1-day Vas leg pain after surgery. (IV) 1-week Vas back pain after surgery. (V) 1-week Vas leg pain after surgery. (VI) Follow-up of Oswestry Disability Index (ODI). (VI) length of hospital stay. (VII) cost-effectiveness.

Study design: A prospective, randomized, controlled trial (RCT) comparing different anesthesia methods should be implemented. This design minimizes bias and ensures that the results can be generalized to a broader population.

### Factors for anesthesia choice

The choice of anesthesia method for full-endoscopic lumbar discectomy should be based on several factors, including patient factors, surgical factors, and anesthesiologist factors.

Patient factors: (I) Patients health status: a spectrum of medical conditions, including pulmonary and cardiovascular diseases, diabetes mellitus, hypertension, obesity, and neurological disorders, introduces a myriad of complexities into the anesthetic management. These conditions alter the physiological landscape in which anesthetic agents operate, demanding meticulous adjustments in the anesthetic strategy. (II) Patient age: older patients might have higher risks with general anesthesia due to age-related physiological changes and potentially more comorbidities. Local or epidural anesthesia might be preferred. (III) Anxiety and Cooperation: the patient’s psychological state is an important factor. Some patients might feel too anxious with the idea of being awake during the procedure and therefore may prefer general anesthesia. On the other hand, a cooperative patient might do well with local or epidural anesthesia. (IV) Pain Tolerance: pain threshold could influence the choice between general and local anesthesia. Some patients might be able to tolerate the procedure under local anesthesia, while others might need general anesthesia. (V) History of Chronic Opioid Use: this condition manifests as a reduced responsiveness to analgesic agents, thereby necessitating an escalation in dosage or a shift to alternative anesthetics to achieve the desired analgesic effects. (VI) Patient’s preference: some patients may have a strong fear or anxiety about certain types of anesthesia, which could also influence the anesthesiologist’s decision.

Anesthesiologist factors: (I) Experience and expertise: certain techniques may require specialized training or familiarity. For instance, successfully implementing spinal or epidural anesthesia requires a deep understanding of the relevant anatomy and technical skills. (II) Patient’s health status: anesthesiologists will evaluate patient’s health status and help choose suitable anesthesia. For instance, patients with a history of lumbar surgery and patients with spinal deformities can make administering spinal anesthesia challenging. Cardiovascular complications and respiratory complications can present challenges for the administration of general anesthesia.

Surgical factors: (I) Surgeons factors: although surgeons do not directly choose the type of anesthesia, their expertise and understanding of the surgical requirements, along with their knowledge of the patient’s condition, can greatly influence the choice of anesthesia. For inexperienced spine endoscopy surgeons, anesthesia methods with intraoperative instant feedback enable less nerve damage. (II) Complexity and expected duration of the surgery: if the surgery is expected to be complex or lengthy, such as in cases of patients with spinal stenosis, spinal deformities, or those requiring reoperation after foraminoscopy, general anesthesia might be chosen.

## Conclusion

Overall, the choice of anesthesia method for full-endoscopic lumbar discectomy should be made on a case-by-case basis, considering the individual patient and surgical factors, as well as the anesthesiologist’s experience and patient preferences.

It is important for surgeons to make the most suitable anesthesia method among all available options for patients. The decision on which anesthesia to use will be made by the patient and their surgeon, taking into consideration all relevant factors.

## Author contributions

BZ conceived the study and wrote the paper. CG, SX, HaoL, and YW modified the manuscript. HaiL supervised the whole process and helped to the solve discrepancy. All authors have read and agreed to the final version of the manuscript.

## Conflict of interest

The authors declare that the research was conducted in the absence of any commercial or financial relationships that could be construed as a potential conflict of interest.

## Publisher’s note

All claims expressed in this article are solely those of the authors and do not necessarily represent those of their affiliated organizations, or those of the publisher, the editors and the reviewers. Any product that may be evaluated in this article, or claim that may be made by its manufacturer, is not guaranteed or endorsed by the publisher.
